# p53 Inhibition Protects against Neuronal Ischemia/Reperfusion Injury by the p53/PRAS40/mTOR Pathway

**DOI:** 10.1155/2021/4729465

**Published:** 2021-12-03

**Authors:** Jianlan Zhao, Yinhui Dong, Xingyu Chen, Xiao Xiao, Bo Tan, Gong Chen, Jin Hu, Dashi Qi, Xiaomu Li, Rong Xie

**Affiliations:** ^1^Department of Neurosurgery, Huashan Hospital, Shanghai Medical College, Fudan University, Shanghai 200040, China; ^2^Center for Clinical Research and Translational Medicine, Department of Neurology, Yangpu Hospital, Tongji University School of Medicine, Shanghai, China; ^3^Department of Endocrinology, Zhongshan Hospital, Fudan University, 180 Fenglin Road, Shanghai 200032, China; ^4^Neurosurgical Institute of Fudan University, Shanghai 200040, China; ^5^Shanghai Clinical Medical Center of Neurosurgery, Shanghai 200040, China; ^6^Shanghai Key Laboratory of Brain Function and Restoration and Neural Regeneration, Shanghai 200040, China

## Abstract

The underlying mechanisms of cerebral ischemia/reperfusion (I/R) injury are unclear. Within this study, we aimed to explore whether p53 inhibition exerts protective effects via the p53/PRAS40/mTOR pathway after stroke and its potential mechanism. Both an in vitro oxygen-glucose deprivation (OGD) model with a primary neuronal culture and in vivo stroke models (dMCAO or MCAO) were used. We found that the infarction size, neuronal apoptosis, and autophagy were less severe in p53 KO mice and p53 KO neurons after cerebral I/R or OGD/R injury. By activating the mTOR pathway, p53 knockdown alleviated cerebral I/R injury both in vitro and in vivo. When PRAS40 was knocked out, the regulatory effects of p53 overexpression or knockdown against stroke disappeared. PRAS40 knockdown could inhibit the activities of the mTOR pathway; moreover, neuronal autophagy and apoptosis were exacerbated by PRAS40 knockdown. To sum up, in this study, we showed p53 inhibition protects against neuronal I/R injury after stroke via the p53/PRAS40/mTOR pathway, which is a novel and pivotal cerebral ischemic injury signaling pathway. The induction of neuronal autophagy and apoptosis by the p53/PRAS40/mTOR pathway may be the potential mechanism of this protective effect.

## 1. Introduction

Cerebral ischemia/reperfusion (I/R) injury is a pathophysiological process that impairs neuronal survival after stroke [1], but its underlying mechanisms are still not clear. p53, a pivotal tumor suppressor, is also thought to exacerbate brain I/R injury [2], although some controversy remains. Previously, we found that inhibition of p53 had protective effects against neuronal oxygen and glucose deprivation (OGD) injury in vitro by activating the mammalian target of rapamycin (mTOR) pathway [3]. The mTOR pathway participates in a variety of physiological processes, including cell metabolism, growth, differentiation, development, and cell survival [4]. Moreover, it is involved in protection against cerebral ischemia [5]. In our previous studies, we demonstrated that the activation of mTOR alleviated stroke-related neuronal injury [6] [7]. However, the specific regulatory mechanisms linking the p53 and mTOR pathways need to be further explored [8].

Proline-rich Akt substrate of 40 kDa protein (PRAS40) is located downstream of the Akt pathway and is also a crucial component of mTOR complex 1 (mTORC1) [9]. It was reported that phosphorylated PRAS40 (pPRAS40) activated mTOR pathways [10]. Similarly, Wang et al. also stated that the ratio between pPRAS40 and PRAS40 determines the activation of mTOR [11]. We demonstrated that PRAS40 knockout mice suffered more severe cerebral I/R injury than wild-type mice, and overexpression of pPRAS40 alleviated cerebral I/R injury and autophagy by activating mTOR [12]. In addition, Havel et al. reported a negative feedback relationship between p53 and PRAS40, suggesting that p53 can be inhibited by its downstream factor PRAS40 [13]. Thus, p53, PRAS40, and mTOR may be intrinsically related and play crucial roles after stroke.

In this study, we proposed a potential mechanism in which p53, PRAS40, and mTOR protect against neuronal I/R injury. We designed in vivo and in vitro experiments to confirm our hypothesis and to explore whether p53 inhibition exerts protective effects via the potential p53/PRAS40/mTOR pathway.

## 2. Materials and Methods

### 2.1. Ethics Statement

All animal experiments were approved by the ethics committee of Huashan Hospital Fudan University and were strictly conducted according to ARRIVE protocols.

### 2.2. Focal Cerebral Ischemia in Rats and Mice

Animals were housed under a 12 : 12 hr light/dark cycle with food and water available ad libitum. In male Sprague-Dawley rats (280 to 320 g), focal cerebral ischemia was generated by 30 mins occlusion of the bilateral common carotid arteries (CCAs) with permanent occlusion of the distal middle cerebral artery as described previously [6]. In male C57BL/6 mice (25 to 30 g), the transient MCA suture occlusion model, induced by middle cerebral artery occlusion (MCAO) for 60 mins, was used, too [12]. Core body temperature was monitored with a rectal probe and kept at 37°C throughout the experiment. Blood gas, heart rate, respiratory rate, and temperature were monitored throughout the surgery and kept within physiological ranges.

### 2.3. Infarction Size Measurement

Infarction was measured by 2,3,5-triphenyl-2H-tetrazolium chloride (TTC) or cresyl violet (CV) staining 48 hrs after stroke onsets as described previously [14] [15]. The area of the infarcted cortex was measured by a person who was blinded to the animal's condition, normalized to the contralateral or bilateral cortex, and expressed as a percentage.

### 2.4. Hematoxylin-Eosin (HE) and TdT-Mediated dUTP Nick-End Labeling (TUNEL) Staining

Protocols for HE [16] and TUNEL [17] staining have been described previously (see in Supplementary Methods). Histological evaluations were performed with HE staining for assessment of neuronal damage. The number of TUNEL-positive cells is counted by an unbiased stereological method [18].

### 2.5. Creation of p53 KO Mice and PRAS40 KO Mice and Genotyping

p53 heterozygote (+/−) and homozygote (−/−) mice originated from C57BL/6 were commercially constructed and fostered by Shanghai Model Organisms, China, and polymerase chain reaction (PCR) was used to analyze the genotyping of wide type (WT, p53^+/+^), p53 heterozygote (p53^+/−^), and p53 homozygote (p53^−/−^) mice. The procedure of PCR is briefly described in Supplementary Methods.

PRAS40 KO mice were kindly provided by Dr. Richard Roth, Department of Chemical and Systems Biology, Stanford University, which were generated using standard homologous recombination methods as our previously studies [12]. PRAS40 KO in C57BL/6 mice was confirmed by performing western blotting analysis.

### 2.6. Behavioral Testing

The neurological score was evaluated 48 hrs after focal cerebral ischemia injury was induced according to a neurological grading score, from 0 (no observable neurological deficit) to 4 (unable to walk spontaneously and a depressed level of consciousness) [19]. The evaluator was blinded to genotypes and experimental treatment.

### 2.7. Lentiviral Vector Construction, Generation, and Titration

We commercially constructed lentiviral vectors containing PRAS40 shRNA (PRAS40 shRNA: 15672, Addgene, Cambridge, MA) to inhibit the expression of PRAS40 and p53 shRNA (p53 shRNA: 12089, Addgene, Cambridge, MA) to inhibit p53 expression, and a scrambled shRNA (Scramble shRNA:1864, Addgene, Cambridge, MA) was used as a control. For p53 overexpression, the p53 cDNA was cloned from the plasmid (p53: 12136, Addgene, Cambridge, MA, USA) into the lentiviral backbone pHR'tripCMV-IRES-eGFP, which contains a CMV promoter and an IRES sequence between its multiple cloning site (MCS) and eGFP as previously described [7] [14]. The IRES sequence enables the independent expression of both the target gene and the eGFP simultaneously. A lentiviral plasmid backbone containing only eGFP was used as a control vector.

We used a 3-plasmid system for lentivirus or adenoviral packaging as detailed in our previous study [3] [7]: the lentiviral transfer vector (pHR'tripCMV–IRES–eGFP) that contains the coding region of various targeted genes as described above; the packaging plasmid (p-delta) that provides all vector proteins driven by the trip CMV promoter, except the envelope protein; and the envelope-encoding plasmid (p-VSVG) that encodes the heterologous vesicular stomatitis virus envelope protein (VSVG) [20]. The procedure is briefly described in Supplementary Methods.

### 2.8. In Vivo Drugs and Lentiviral Vector Injection

Drugs and viral vectors were coded to blind the surgeon performing both the virus injections and stroke models as previous study [7]. Rapamycin (Calbiochem, Billerica, MA, USA) was dissolved in PBS to a final concentration of 0.1 mM. 10 *μ*l of rapamycin was infused into the ventricular space ipsilateral to the ischemia using a microsyringe pump controller 1 hr prior to ischemia [14]. Lentiviruses were injected by a 10 *μ*l needle into the left cortex 5 days prior to ischemia [6].

### 2.9. Primary Neuronal Culture

Primary neuronal cultures were prepared using timed-pregnant Sprague-Dawley rats (E18, Charles River Laboratories International, Wilmington, MA) as previously reported [12]. This procedure is described in Supplementary Methods.

### 2.10. In Vitro Oxygen-Glucose Deprivation and Reperfusion (OGD/R) Model, Gene Transfer, and Cell Viability Assay

Primary mixed neuronal cultures were prepared from rat fetal brains and experiments were performed days 9 to 11 after preparation. OGD was induced for 6 hrs in a hypoxic chamber as previously described [6]. Methods of OGD and reperfusion treatment are briefly described in Supplementary Methods. Cell viability was quantified by measuring lactate dehydrogenase (LDH) release 6 hrs after OGD restoration using a previously described colorimetric assay [21] (Supplementary Methods).

### 2.11. Protein Preparation In Vivo and In Vitro and Western Blotting Procedure

Ischemic brains corresponding to ischemic core were harvested at 48 hrs after stroke onset, and brain tissues underwent sham surgery without ischemia was also prepared for western blotting. Whole-cell protein was extracted from the fresh brain tissues. In vitro, cells were harvested at 48 hrs after gene transfer, and homogenized in the cold cell extraction buffer containing 1 mmol/l phenylmethylsulphonyl fluoride and the protease inhibitor cocktail. The homogenate was centrifuged at 10392 g (13000 rpm) for 20 mins at 4°C, and the supernatant was removed for protein detection. Both in vivo and in vitro, protein concentrations were measured using the Bradford assay before western procedure, and western blotting was performed as described with modification [22] (Supplementary Methods). The manufacturers and catalog numbers of all primary antibodies used are listed in Supplementary Table S1.

### 2.12. Immunofluorescence Staining and Confocal Microscopy

Immunofluorescent staining of brain sections [14] and confocal microscopy [7] was performed as previously described. This information was provided in Supplementary Methods. The manufacturers and catalog numbers of all primary antibodies used are listed in Supplementary Table S1.

### 2.13. Transmission Electron Microscopy (TEM)

TEM was performed as described in Supplementary Methods. Briefly, mice were first perfused with PBS, then perfused with 4% paraformaldehyde PFA and 1% glutaraldehyde for 4 h, and finally fixed in 1% osmium tetroxide and 2% uranyl acetate. After dehydration of ethanol and propylene oxide, the tissue was embedded in pure, fresh Quetol-812 epoxy resin. Then, sections of the brain were stained with 2% uranylacetate and 0.3% lead citrate and then were analyzed on a FEI at 80 kV.

### 2.14. Statistical Analysis

Data are expressed as mean ± SEM. Differences were considered statistically significant for *p* < 0.05. Student's *t*-tests were used when 2 groups were compared. One-way or two-way ANOVA was used followed by the Fisher least significant difference post hoc test using Prism 7 (GraphPad Software for Science, San Diego, CA). All assessments were by blinded observers.

## 3. Results

### 3.1. p53 Aggravated Neuronal Ischemic/Reperfusion Injury by Inhibiting the mTOR Pathway Both In Vitro and In Vivo

**Figure 1:**
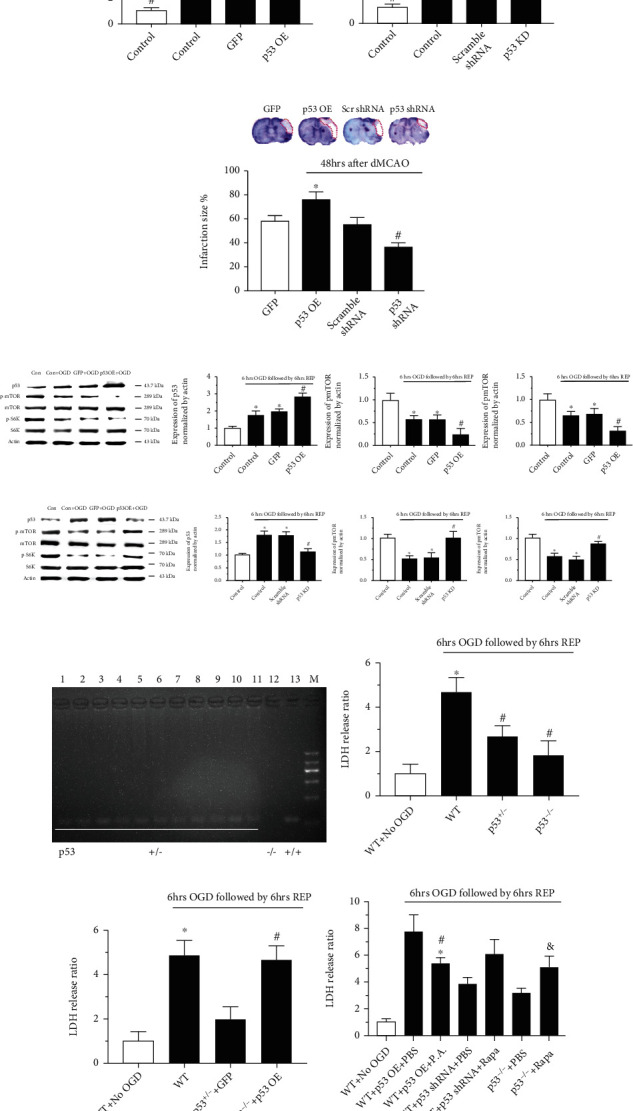
p53 exacerbated cerebral I/R injury by inhibiting the mTOR pathway both in vitro and in vivo. (a) In primary cultured rat neurons after OGD/R injury (6 h OGD followed by 6 h reperfusion), the LDH level increased in the p53-overexpressing group (p53 OE+OGD) compared with the control groups (control+OGD and GFP+OGD). In the p53 knockdown group (p53 KD+OGD), LDH release decreased compared with that in the control groups (control+OGD and scramble shRNA+OGD). ^∗^*p* < 0.05 vs. the control+OGD group; ^#^*p* < 0.05 vs. the GFP+OGD or scramble shRNA+OGD group. (b) Forty-eight hours after dMCAO in SD rats, the infarction size, normalized to the contralateral cortex, greatly increased in the p53-overexpressing group (p53 OE); however, administration of p53 shRNA (p53 shRNA group) reduced the total infarction size. ^∗^*p* < 0.05 vs. the GFP group; ^#^*p* < 0.05 vs. the scramble shRNA group. (c, d) In the p53-overexpressing group, the expression levels of p-mTOR and p-S6K decreased compared to those in the control groups (control+OGD and GFP+OGD). In the p53 knockdown group, the levels of p-mTOR and p-S6K obviously increased compared to those in the control groups (control+OGD and scramble shRNA+OGD). ^∗^*p* < 0.05 vs. the control+OGD group; ^#^*p* < 0.05 vs. the GFP+OGD or scramble shRNA+OGD group. *n* = 14–21/group in at least 3 independent experiments. (e) Confirmation of p53 gene KO in mice. PCR for genotyping p53 KO mice. Lane M: DNA size marker; lanes 1 to 11: C57 heterozygote (+/−); lane 12: homozygote (-/-); lane 13: homozygote (+/+). (f) The LDH release ratio after 6 hrs OGD followed by 6 hrs reperfusion was much higher in primary cultured WT mice neurons than that in p53^+/-^ or p53^−/−^ mice. ^∗^*p* < 0.05 vs. WT+No OGD; ^#^*p* < 0.05 vs. WT+OGD. (g) In primary cultured p53^−/−^ mice neurons, when p53 was overexpressed (OE), the LDH level greatly increased compared to control group (p53^−/−^ + GFP). ^∗^*p* < 0.05 vs. WT+No OGD; ^#^*p* < 0.05 vs. p53^−/−^ + GFP. (h) Inhibition of p53 reduced the LDH level via activating the mTOR pathway. The LDH level can be reduced by administrating phosphatidic acid (an agonist of mTOR pathway, P.A.) when p53 was overexpressed. Similarly, when p53 shRNA was administrated, rapamycin (an antagonist of mTOR pathway, Rapa) could increase the LDH release ratio. Moreover, in primary cultured p53^−/−^ mice neurons, the LDH release was more severe when treated with Rapa compared with PBS. ^∗^*p* < 0.05 vs. WT+p53 OE+PBS; ^#^*p* < 0.05 vs. WT+p53 shRNA+PBS; ^&^*p* < 0.05 vs. p53^−/−^+PBS. *n* = 14-21/group in at least 3 independent experiments.

We first characterized the effects of p53 overexpression (OE) and knockdown (KD) against ischemic injury in primary cultured neurons and SD rats (Figures 1(a) and 1(b)). After 6 h of OGD followed by 6 h of reperfusion in vitro, LDH release increased in the p53 OE cells but decreased in the p53 KD cells (Figure 1(a)). In vivo, following administration of the p53 gene or shRNA, the infarction size was significantly increased or decreased, respectively, at 48 h after dMCAO (Figure 1(b)). We then measured the levels of phosphorylated mTOR and S6K1 after oxygen glucose deprivation and reperfusion (OGD/R) injury in vitro. The results showed that p-mTOR and p-S6K levels were decreased by p53 OE (Figure 1(c)) but increased by p53 KD (Figure 1(d)) after OGD/R injury. The total protein levels of mTOR and S6K1 were relatively stable.

To next evaluate whether inhibition of p53 alleviated hypoxia-related injury, we constructed homozygous p53 knockout (KO) mice (p53^−/−^) and obtained heterozygous (p53^+/−^) mice by mating with WT mice (p53^+/+^) (Figure 1(e)). In vitro, p53 KO (p53^−/−^) or p53 heterozygosity (p53^+/−^) reduced LDH release in primary cultured neurons subjected to OGD/R injury (Figure 1(f)), but the protective effects were abolished by p53 OE using a lentiviral p53 vector (Figure 1(g)).

Similarly, OGD/R injury of primary cultured neurons from WT mice was exacerbated after p53 OE. However, when PA (an mTOR agonist) was administered, ischemic injury was alleviated (Figure 1(h)). In addition, when primary WT mouse neurons were transfected with p53 shRNA, LDH levels decreased. When rapamycin, an mTOR inhibitor, was simultaneously administered, OGD/R injury worsened. Moreover, the application of rapamycin worsened OGD/R injury in primary p53 KO mouse neurons (Figure 1(h)).

**Figure 2:**
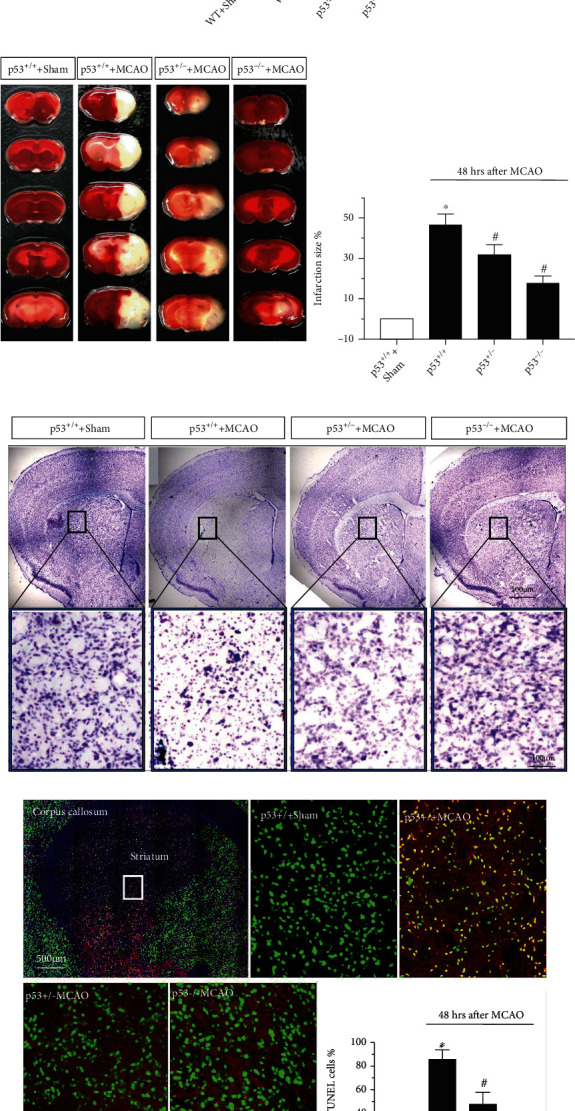
Neurological deficits, infarction size, and neuronal apoptosis were less severe 48 h after MCAO in p53 KO rats. (a) The neurological deficit scores were significantly lower in p53^−/−^ mice 48 hrs after MCAO. Compared with WT mice, the neurological deficit scores were lower in p53^+/-^ mice and much lower in p53^−/−^ mice. ^∗^*p* < 0.05 vs. WT+sham; ^#^*p* < 0.05 vs. WT+MCAO. *n* = 6-8/group. (b) The infarction size normalized to the bilateral cortex was smaller in p53^−/−^ and p53^+/-^ mice than in p53^+/+^ mice 48 h after MCAO. ^∗^*p* < 0.05 vs. the p53^+/+^+sham group; ^#^*p* < 0.05 vs. the p53^+/+^+MCAO group. (c) The analyzed samples were obtained from ischemic brains of mice 48 h after MCAO. After HE staining (×100), more normal neurons were observed in p53 KO and heterozygous mice than in p53 WT mice, which suggested less severe cerebral I/R injury. (d) By performing TUNEL staining in the ischemic brains of mice after MCAO (×400), positive apoptotic cell counts (red) were quantified. The number of positive TUNEL cells was significantly lower in p53^+/-^ and p53^−/−^ mice than in p53^+/+^ mice. ^∗^*p* < 0.05 vs. the p53^+/+^+sham group; ^#^*p* < 0.05 vs. the p53^+/+^+MCAO group. *n* = 6–8/group.

Moreover, an in vivo study confirmed that both p53 KO and heterozygosity improved neurological behavioral deficits (Figure 2(a)) and mitigated infarct sizes at 48 h after MCAO, but p53 KO showed a stronger protective effect than p53 heterozygosity (Figure 2(b)). Additionally, HE and TUNEL staining indicated that p53 KO and heterozygous mice had more slight ischemic damage after MCAO than p53 WT mice (Figures 2(c) and 2(d)).

**Figure 3:**
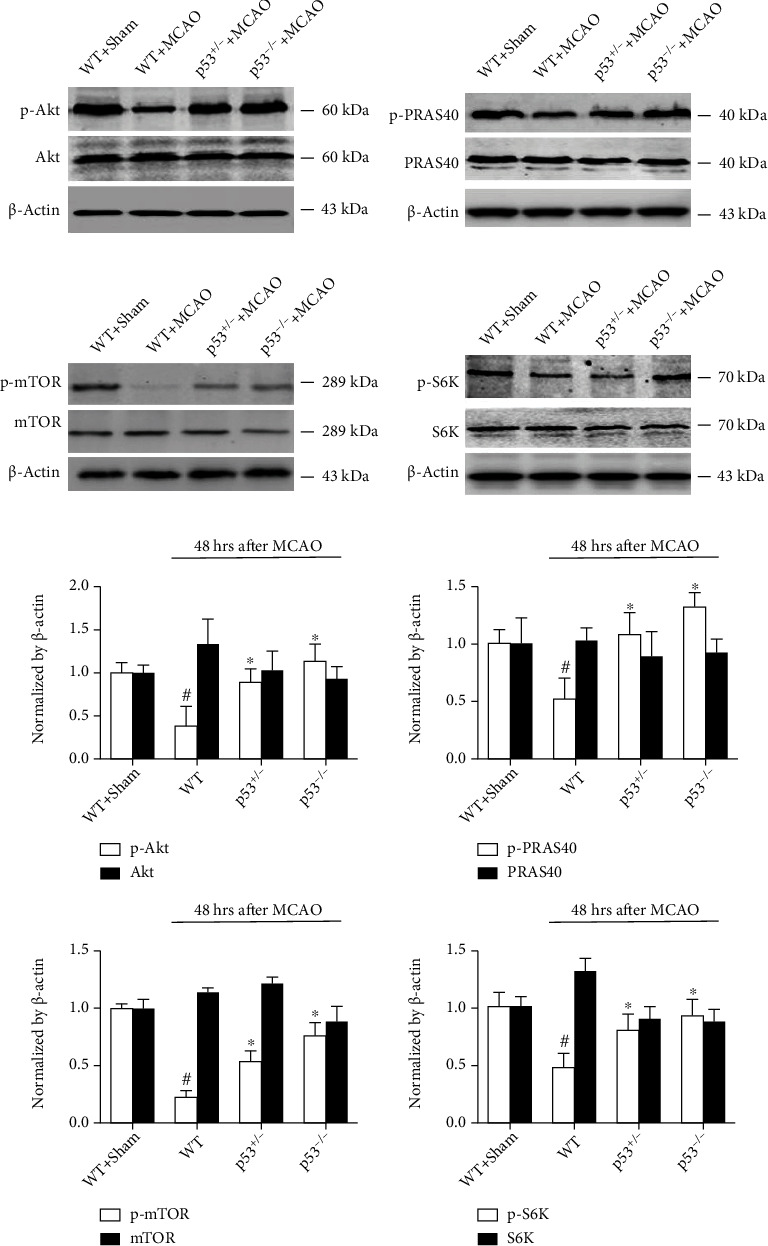
Proteins related to Akt/PRAS40/mTOR were examined by western blotting (a) and quantified (b) 48 h after MCAO. The expression of p-Akt, p-PRAS40, p-S6K, and p-mTOR was higher in p53^−/−^ and p53^+/-^ mice than in p53^+/+^ mice (WT). ^∗^*p* < 0.05 vs. the WT+MCAO group; ^#^*p* < 0.05 vs. the WT+sham group. *n* = 6–8/group.

The effects of p53 KO or heterozygosity on the protein levels of p-Akt, p-PRAS40, p-mTOR, and p-S6K in ischemic brain tissues 48 h after MCAO were examined and quantified using western blotting (Figures 3(a) and 3(b)). p53 KO increased the levels of all these phosphorylated proteins after MCAO. Additionally, the p53^+/−^ mice showed significantly upregulated protein levels of p-Akt, p-PRAS40, p-mTOR, and p-S6K after MCAO compared with the p53 WT mice. Nevertheless, neither p53 KO nor p53 heterozygous mice showed altered total protein levels compared with p53 WT mice after sham surgery or MCAO.

### 3.2. The Effects of p53 Overexpression and Knockdown against OGD/R Injury Were Abolished in Primary Cultured PRAS40 KO Mouse Neurons

**Figure 4:**
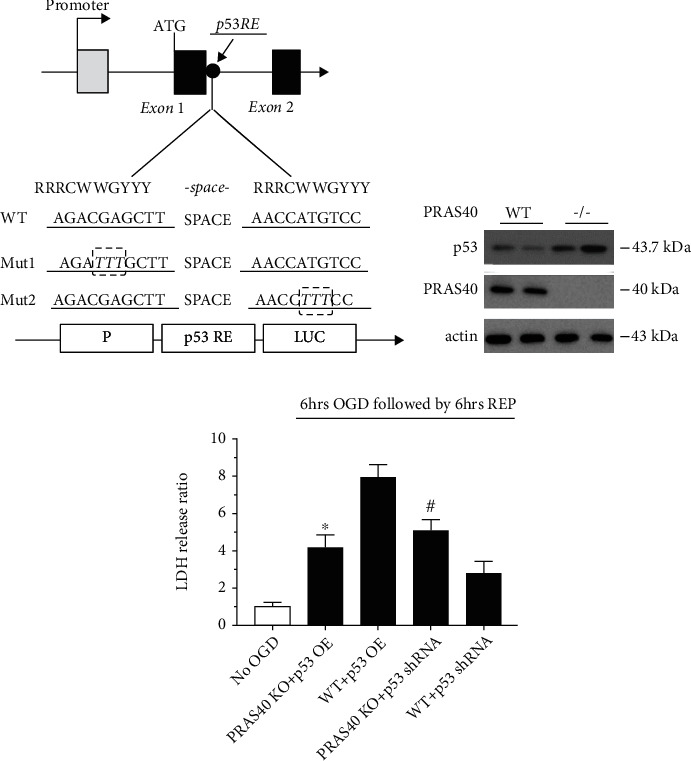
PRAS40 was located downstream of p53. (a) By using bioinformatics analysis, p53 RE sequences were identified in the PRAS40 gene promoter, which indicated that PRAS40 was regulated by p53. (b) The expression of p53 obviously increased in the PRAS40^−/−^ mouse brain. (c) LDH release after OGD/R injury in primary cultured PRAS40 KO mouse neurons was greater than that in WT mouse neurons, even though p53 was overexpressed. Moreover, when p53 was knocked down, OGD/R injury was worse in PRAS40 KO mice than in WT mice. ^∗^*p* < 0.05 vs. the WT+p53 OE group; ^#^*p* < 0.05 vs. the WT+p53 shRNA group. *n* = 14–21/group in at least 3 independent experiments.

After demonstrating that the mTOR signaling pathway was activated by inhibiting p53 as well as in p53 KO and heterozygous mice (Figure 1 to Figure 3), we tried to clarify the potential mechanism linking p53 and the mTOR pathway. Since our previous studies suggested that PRAS40 had a regulatory effect on mTOR [12], we explored the relationship between p53 and PRAS40. First, p53 reacting element (RE) sequences were identified in the area of the PRAS40 gene promoter using bioinformatics analysis (Figure 4(a)). Western blotting confirmed that the expression of p53 was significantly higher in the brain tissue of PRAS40 KO mice than in that of PRAS40 WT mice (Figure 4(b)). Moreover, we investigated the effects of p53 gene transfer on neuronal death induced by OGD/R injury in vitro. We measured LDH release after 6 h of OGD followed by 6 h of reperfusion using primary cultured PRAS40 KO and WT mouse neurons with p53 OE and KD. The results showed that when p53 was overexpressed in primary cultured neurons, the LDH level was significantly lower in neurons from PRAS40 KO mice than in those from PRAS40 WT mice. Furthermore, following transfection with p53 shRNA lentivirus vector, OGD/R injury in primary cultured PRAS40 WT mouse neurons was less serious than that in PRAS40 KO mouse neurons (Figure 4(c)). These findings suggested that PRAS40 gene knockout eliminated both the p53 OE-induced exacerbation of OGD/R injury and the p53 KD-induced alleviation of OGD/R injury in primary cultured neurons. Thus, PRAS40 might be a potential target of p53 in regulating the mTOR pathway.

### 3.3. PRAS40 KD Blocked the Protective Effects of p53 KO against Cerebral Ischemic Injury by Increasing Neuronal Apoptosis and Autophagy and Inhibiting the mTOR Pathway

**Figure 5:**
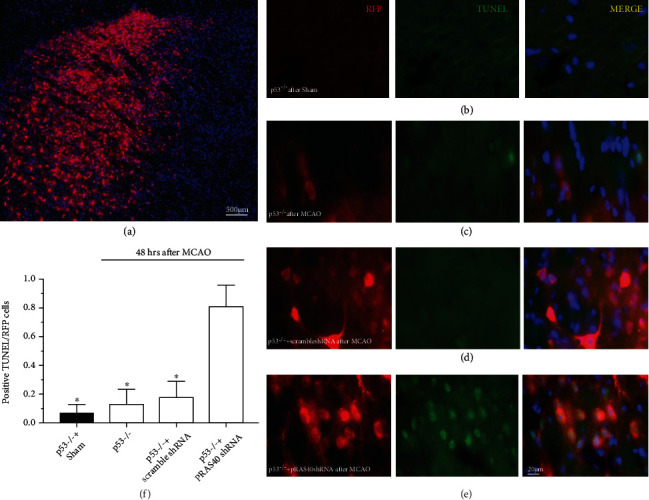
The TUNEL staining in primary cultured p53^−/−^ mice neurons. (a) The TUNEL staining in primary cultured p53^−/−^ mice neurons administrated with PRAS40 shRNA after MCAO (×100). 48 hrs after MCAO in p53^−/−^ mice neurons, the positive TUNEL/RFP cells ratio was significantly higher when pre-transfected with PRAS40 shRNA (e, ×400) than none pretransfected neurons (b, c; ×400) or pretransfected with scramble shRNA (d, ×400). (f) Quantification. ^∗^*p* < 0.05 vs. p53^−/−^+PRAS40 shRNA. *n* = 6-8/group.

**Figure 6:**
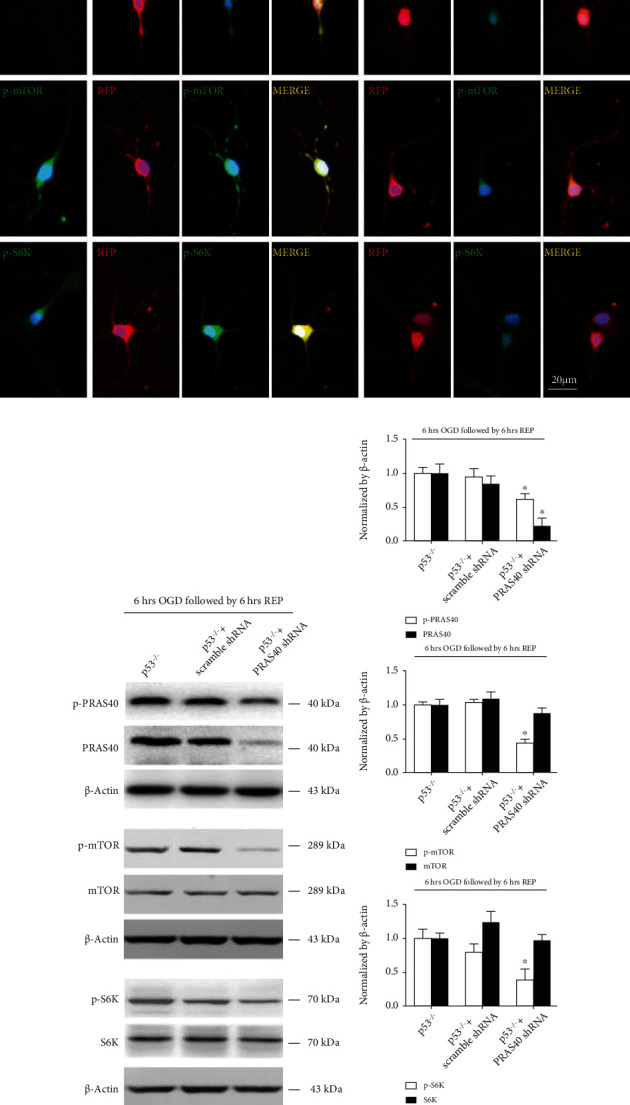
PRAS40 knockdown inhibited the activities of the mTOR pathway. As shown by immunostaining (a, ×400) and western blotting (b, bands; c, quantification), in primary cultured p53^−/−^ mouse neurons subjected to OGD, the expression of p-mTOR and p-S6K was distinctly reduced after pretransfection with PRAS40 shRNA compared with that in neurons pretransfected with or without scramble shRNA. ^∗^*p* < 0.05 vs. the p53^−/−^+scramble shRNA group. *n* = 14–21/group in at least 3 independent experiments.

We used TUNEL staining to confirm that PRAS40 shRNA transfection significantly increased neuronal apoptosis after MCAO in p53 KO mice compared to scramble shRNA transfection or no transfection (Figure 5). Then, we detected mTOR-related protein expression after OGD/R injury using immunostaining and western blotting in primary cultured p53 KO mouse neurons with or without PRAS40 shRNA transfection (Figure 6). After OGD/R injury, the protein levels of PRAS40, p-mTOR, and p-S6K remained similar in p53 KO mouse neurons and neurons pretransfected with scramble shRNA but decreased in p53 KO mouse neurons pretransfected with PRAS40 shRNA, which suggested that PRAS40 KD blocked the protective effects of p53 KO against OGD/R injury and increased neuronal apoptosis in vitro by inhibiting the mTOR pathway.

**Figure 7:**
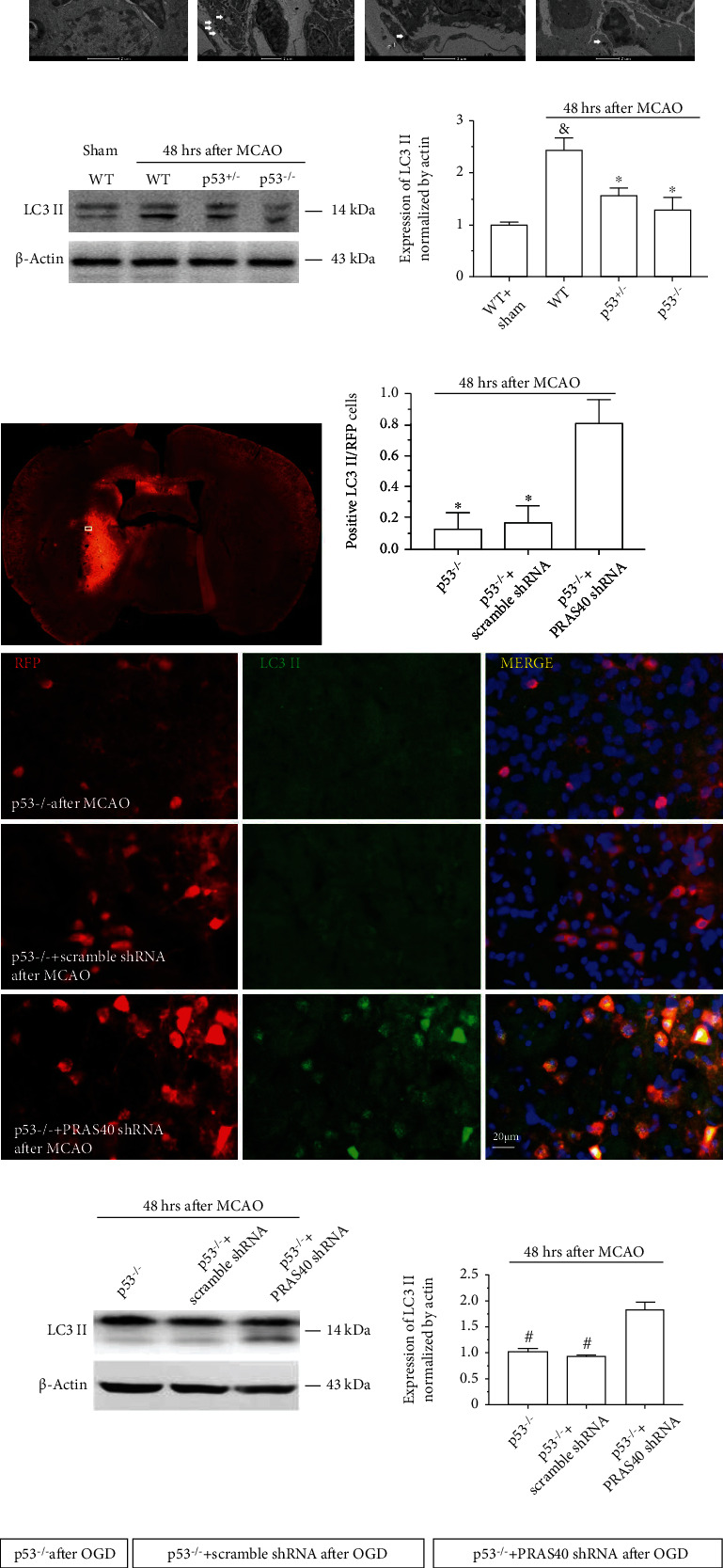
Neuronal autophagy was exacerbated by PRAS40 knockdown both in vivo and in vitro. (a) As shown by immunostaining (×100) and TEM in ischemic mouse brains 48 h after MCAO, more LC3 II-positive cells (immunostaining) and autophagosomes (TEM; white arrow) were observed in p53^+/+^ WT mice than in p53^+/-^ or p53^−/−^ mice. (b) In the ischemic brain 48 h after MCAO, the level of LC3 II was significantly higher in WT mice than in p53^+/-^ or p53^−/−^ mice. ^∗^*p* < 0.05 vs. the WT+MCAO group; ^&^*p* < 0.05 vs. the WT+sham group. (c) As shown by immunostaining (×400) of p53^−/−^ mouse brains 48 h after MCAO, the level of LC3 II and the ratio of LC3 II-positive to RFP-positive cells were much higher in cells pretransfected with PRAS40 shRNA than in those pretransfected with or without scramble shRNA. ^∗^*p* < 0.05 vs. the p53^−/−^+PRAS40 shRNA group. (d) In p53^−/−^ mouse brains 48 h after MCAO, PRAS40 shRNA administration increased the level of LC3 II much more than that in the control groups treated with or without scramble shRNA. ^#^*p* < 0.05 vs. the p53^−/−^+PRAS40 shRNA group. *n* = 6–8/group. (e) As shown by immunostaining (×400), the expression of LC3 II in primary cultured p53^−/−^ mouse neurons were markedly higher when cells were pretransfected with PRAS40 shRNA than when they were pretransfected with or without scramble shRNA.

Moreover, electron microscopy (Figure 7(a)) and western blotting (Figure 7(b)) showed that in both p53 KO and heterozygous mice, the expression of LC3 II was significantly lower than that in p53 WT mice after MCAO. In addition, immunostaining and western blotting demonstrated that the LC3 II level significantly increased when PRAS40 was knocked down in p53 KO mice after MCAO (Figures 7(c) and 7(d)) and in neurons from p53 KO mice after OGD/R injury (Figure 7(e)). These results indicated that PRAS40 knockdown independently exacerbated neuronal autophagy after cerebral ischemic injury in vitro and in vivo even when p53 was knocked out.

## 4. Discussion

This study provides the first evidence that inhibition of p53 alleviates neuronal I/R injury both in vivo and in vitro via the pivotal p53/PRAS40/mTOR pathway.

### 4.1. PRAS40 Connects p53 and the mTOR Pathway to Protect against Neuronal I/R Injury

The mTOR pathway is involved in brain injury induced by ischemia. Although some studies have suggested that the activity of mTOR is detrimental [23], there is evidence that upregulation of Akt/mTOR activity is neuroprotective against ischemic brain injury [24], which is in accordance with our previous studies [6] [14]. Thus, an appropriate regulatory mechanism to induce neuronal protective effects through the mTOR pathway is required.

I/R injury, including in the myocardium, kidney, and brain, can be affected by the activity of p53, but the role of p53 is still controversial. p53 was shown to have neuroprotective and anti-inflammatory effects after ischemic kidney injury [25] [26] but was also shown to exacerbate cerebral I/R injury [27]. Therefore, p53 may exert opposite effects on I/R injury due to its involvement in various signaling pathways. To the best of our knowledge, studies concerning the effects of p53 and mTOR on brain ischemic injury are rare. Myocardial I/R injury may be related to the expression of p53 and activities of the mTOR pathway [28]. Similarly, we previously found that inhibition of p53 protected against cerebral I/R injury in vitro via mTOR [3], which was also confirmed by this study and other works [29]. p53/mTOR may be a potential regulatory pathway against cerebral I/R injury. Since bioinformatics analysis and a literature review indicated that no direct regulatory loci of p53 were detected in the mTOR sequence, we assumed that there may be a connection between p53 and mTOR. Previously, we found that PRAS40 linked the Akt and mTOR pathways and played a pivotal role in protecting against stroke [12]; moreover, it was suggested by bioinformatics analysis that p53 RE sequences were identified in the PRAS40 gene promoter. Thus, we hypothesized that PRAS40 might be the link between p53 and the mTOR pathway.

### 4.2. p53/PRAS40/mTOR May Be a Novel and Pivotal Cerebral Ischemic Injury Signaling Pathway

The p53/PRAS40/mTOR pathway was first proposed and verified by our study, and we also clarified its protective mechanism in cerebral ischemic injury after stroke. As shown in Figure 5(c), OGD/R injury was less severe in PRAS40 KO mouse neurons even when p53 was overexpressed. When p53 was knocked down, PRAS40 KO also did not exacerbate OGD/R injury in neurons. These results indicated that PRAS40 acted as a core molecule located downstream of p53, and cerebral I/R or OGD/R injury was regulated by the activities of p53 via PRAS40. Moreover, we also found that the mTOR pathway was inactivated when PRAS40 shRNA was administered to p53^−/−^ mouse neurons. These findings were consistent with our previous studies [12] and further confirmed that the mTOR pathway could be regulated through PRAS40. Therefore, we hypothesized the existence of the p53/PRAS40/mTOR pathway.

PRAS40 plays a pivotal role in the regulation of the mTOR pathway by p53. In this study, we found that when PRAS40 was knocked down, the mTOR signaling pathway could not be activated or inactivated by p53, and cerebral I/R injury remained relatively stable (PRAS40 KO with p53 overexpression and PRAS40 KO with p53 knockdown, Figure 5(c)). Thus, we concluded that PRAS40 may act as a “bridge” linking p53 and the mTOR pathway.

### 4.3. The p53/PRAS40/mTOR Pathway Regulates Neuronal Autophagy and Apoptosis: A Potential Cerebral Ischemic Injury Mechanism after Stroke

A variety of diseases and pathophysiological processes, including cancer [30] and I/R injury [31], can be promoted by autophagy and apoptosis. p53 is known to be involved in the autophagy process [32]. The interaction between p53 and autophagy is complex. Autophagy suppresses p53, and p53 activates autophagy [32]. In cerebral ischemic injury, p53-induced autophagy may promote neuronal death and exacerbate brain hypoxia injury [33], but these roles remain controversial. Various p53 signaling pathways, including NF-*κ*B/p53 [33], p53-TIGAR [34], and TAF9b/p53 [35], are involved in cell autophagy, but the specific molecular mechanism is still unclear. It was reported that autophagy is promoted by p53/AMPK/mTOR signaling in human glioma U251 cells [36]. Therefore, the p53/mTOR pathway may exacerbate neuronal autophagy in cerebral ischemic injury after stroke.

In addition, p53 participates in the process of apoptosis after ischemic stroke [37]. It was previously reported that brain I/R injury correlates with neuronal apoptosis, which can be regulated by p53 [38]. In various stroke models, p53 deficiency or application of p53 inhibitors significantly attenuated brain damage [39]. Neuronal apoptosis can be regulated by p53 via signaling pathways such as p53/Notch [40] and DAPK1/p53 [41]; however, the specific molecular mechanism of p53-induced apoptosis after brain ischemic injury is not clear. It was also found that neuronal apoptosis can be regulated by the Akt/mTOR pathway [5]. As reported in our previous study, p53 inhibition has a pivotal protective effect against cerebral I/R injury via mTOR signaling [3], suggesting that neuronal apoptosis after brain ischemic injury may be regulated by p53 via the mTOR pathway.

In this study, we hypothesized that neuronal autophagy and apoptosis via the p53/PRAS40/mTOR pathway might be a potential mechanism that worsens ischemic injury after stroke. As to the role of autophagy in the I/R-injured neurons, we believe that its significance is considerable and nonnegligible, based on the following evidence: (1) after stroke models were built either in vivo or vitro, the level of autophagy related protein (LC3-II, by western blotting) was altered, which was reversed by knockout of p53, and knockdown of PRAS40 at least partially abolished this effect; (2) immune-fluorescence assay not only revealed the protein level alteration of LC3-II but also the formation of the autophagosome. We think it is direct evidence to support the role of autophagy in the pathogenesis of I/R injury on neurons; (3) the results of electron microscope also indicated that knockout of p53 suppressed the formation of autophagosome in the MCAO injury of the brain in vivo.

Similarly, as shown by TUNEL staining, neuronal apoptosis after MCAO was alleviated in p53^−/−^ mice, but when PRAS40 shRNA was administered to p53^−/−^ mice, more apoptotic neurons were observed after MCAO. These findings were in accordance with our hypothesis that neuronal autophagy and apoptosis after cerebral ischemic injury is regulated via the p53/mTOR pathway and that PRAS40 is required for autophagy and apoptosis regulation. But the specific underlying mechanisms remain not fully understood, further research is required.

### 4.4. Limitations

First, clinical ischemic brain samples are required to confirm our conclusions. Moreover, a more detailed mechanistic study and bioinformatics analysis will be performed to identify the p53/PRAS40/mTOR pathway. Third, the interaction between p53/PRAS40/mTOR and other signaling pathways should be explored to clearly elucidate the mechanism of neuronal ischemic injury after stroke.

## 5. Conclusions

p53 inhibition protects against neuronal I/R injury after stroke via the p53/PRAS40/mTOR pathway, which is a novel and pivotal cerebral ischemic injury signaling pathway. The induction of neuronal autophagy and apoptosis by the p53/PRAS40/mTOR pathway may be the potential mechanism of this protective effect.

## Data Availability

The datasets used and/or analyzed during the current study are available from the corresponding author, Rong Xie, on reasonable request.

## Abbreviations

CCAs: Common carotid arteries
CV: Cresyl violet
dMCAO: Distal middle cerebral artery occlusion
HE: Hematoxylin-eosin
I/R: Ischemia/reperfusion
KO: Knockout
KD: Knockdown
LDH: Lactate dehydrogenase
mTOR: Mammalian target of rapamycin
mTORC1: mTOR complex 1
MCAO: Middle cerebral artery occlusion
OE: Overexpression
OGD/R: Oxygen and glucose deprivation/reperfusion
PRAS40: Proline-rich Akt substrate of 40 kDa protein
pPRAS40: Phosphorylated PRAS40
TEM: Transmission electron microscopy
TTC: 2,3,5-Triphenyl-2H-tetrazolium chloride
TUNEL: TdT-mediated dUTP nick-end labeling.
