# Supplementary material for: p53 Inhibition Protects against Neuronal Ischemia/Reperfusion Injury by the p53/PRAS40/mTOR Pathway
Source: Oxid Med Cell Longev. 2021 Dec 3;2021:4729465. doi: 10.1155/2021/4729465 (PMC8664552; doi:10.1155/2021/4729465)
Supplement: Supplementary Materials — Supplementary experimental materials and methods. [file 4729465.f1.pdf]

Supplementary Materials

Hematoxylin-eosin (HE) and TdT-mediated dUTP Nick-End Labeling (TUNEL) Staining

All tissue samples were fixed in 4% formalin, decalcified in ethylene diamine tetraacetic acid (EDTA) solution, and embedded in paraffin. They were cut into 4-μm thickness sections and heated in an oven for approximately 60 mins. Then, the sections were deparaffinized with xylene and rehydrated followed by a graded series of ethanol. After heat-induced antigen retrieval and endogenous peroxidase blocking, the sections were incubated with primary antibodies overnight at 4°C. Samples were observed using a CX21 optical microscope (Olympus Optical Co., Ltd, Tokyo, Japan) after sealing.

Forty-eight hrs after onset of stroke, animals were anesthetized and perfused through the heart with cold saline followed by 4% paraformaldehyde in phosphate buffer. Removed brains were fixed in 4% paraformaldehyde overnight and then embedded in paraffin. Brain tissues were cut into 5-μm thick coronal sections, deparaffinized, and rehydrated. For TUNEL staining, sections were processed with an In-Situ Cell Death Detection Kit, AP (Roche, Diagnostics GmbH, Mannheim, Germany). In vitro, post-treatment cells were treated with the highest concentration (640μg/mL) of nanospheres for 24 hrs. After the incubation period, the culture medium was aspirated, and the cell layers were trypsinized. The trypsinized cells were reattached on 0.01% polylysine-coated slides, fixed with 4% methanol-free formaldehyde solution, and stained according to the DeadEnd fluorometric TUNEL system protocol.

TUNEL-positive cells were visualized by light microscopy and quantified in five non-overlapping visual fields for each slice. To determine the percentage of cells undergoing apoptosis, 1000 cells were counted in each experiment.

Polymerase chain reaction (PCR) analysis

Briefly, genomic DNA was extracted from the tail of mice using a DNeasy Blood and Tissue Kit (Qiagen, Germantown, MD, USA), and genomic PCR was performed with Taq DNA Polymerase High Fidelity (Invitrogen, Carlsbad, CA, USA) under the following conditions: 94°C for 60 secs; 30 cycles at 94°C for 60 secs, 55°C for 45 secs, and 72°C for 45 secs; and 72°C for 6 mins. The primer pair for p53 (forward: 5’ACTGCATGGACGATCTGTTG3’, reverse: 5’GTGACAGGGTCCTGTGCTG3’) amplifies a fragment of 225 bp in mice homozygous or heterozygous for endogenous gene (no band will be generated by this oligo pair in a cre-recombinant homozygous mice).

Lentiviral vector generation and titration

Briefly, 293T cells were grown in Dulbecco’s modified Eagle medium (DMEM, Gibco, Grand Island, NY, USA) containing 10% fetal bovine serum (Gibco) and 1% penicillin/streptomycin (Gibco). A mixture of 45μg of transfer vectors, 30μg of packaging plasmids and 15μg of envelope-encoding plasmids was transiently transfected into 3 separate T175 flasks containing 1.5×10^7 HEK-293T cells using the calcium phosphate precipitation (CPP) method. Supernatants were collected 72 hrs post-transfection and viral particles were concentrated by ultracentrifugation. Viruses were resuspended in phosphate-buffered saline (PBS) and kept at −80°C until use. The virus particles were tittered with the TCID50 method. Virus titers ranged from 1×10^8–5×10^8 TU/ml and were diluted in PBS to the final concentration of 1×10^8 TU/ml before gene transfer was conducted.

Primary neuronal cultures

Rats were anesthetized with isoflurane and the E18 embryos were removed. The cortical region of the fetal brains was dissected in warm media and pooled together. The cortices were triturated and incubated in papain for 20 mins at 37°C, then centrifuged at 1500 rpm for 5 mins at room temperature (RT). Cells were resuspended in MEM (minimal essential medium) (Gibco, Grand Island, NY, USA) containing 10% fetal horse serum (Hyclone, Logan, UT, USA), 2 mmol/l glutamine (Gibco), 25 mM glucose, and 1% penicillin/streptomycin (Gibco). Cells were plated onto poly-D-lysine-coated tissue culture plates at 7.5×10^5 cells/ml. Media were completely changed after 24 hrs. One-half medium changes were performed at day 4. Cultures were incubated at 37°C in a 5% CO_2 incubator and experiments were performed after days 9–11.

Oxygen-Glucose Deprivation (OGD) and Reperfusion

Aspirate the former medium in the 6-well plates 9hrs former medium to simulate ischemia; the control should be disposed with BSS5.5 and incubated in 37°C, 5% CO_2. Then place the OGD-REP groups in the Hypoxia Incubator Chamber, and 10-20 mL 2-time sterile H_2O to maintain the proper humidity within the chamber. Lock the clamp and open the inlet of the chamber and link the bacteria filter with the outlet tube of N_2 Bottle. Next, open the switch and modulate the flow volume to 20L/min and sustain the nitrogen flow for 5 mins and then block the outlet and inlet of the chamber to maintain the low O_2 pressure within the chamber. Place the chamber into the 37°C incubator for 6 hrs. and control groups areincubated directly in the 37°C, 5% CO2 incubator.

Fetch the OGD-REP groups and aspirate the former BSS0. Well rinse twice with BSS5.5, and add the same volume of it as former BSS0 into wells. Incubate the OGD-REP and control groups in the 37°C, 5% CO2 incubator for 6 hrs.

Lactate dehydrogenase (LDH) release measurement

100 mL of cell-free supernatant was transferred to 96-well plates. The supernatant was incubated with 150 mL of NADH/phosphate buffer (0.15 mg/mL) for 10 mins. Then, 30 mL of sodium pyruvate (2.97 mg/mL) was added and the absorbance wavelength was measured at 340nm using a microplate reader. Background absorbance was subtracted and the percentage of LDH release was calculated based on an LDH standard curve.

Western blotting

Thirty micrograms of protein in each lane was subjected to SDS-PAGE using 4-15% Ready Gel (catalog #L050505A2; Bio-Rad, Hercules, CA) under 200V for 45 mins. Protein bands were transferred from the gel to polyvinylidinene fluoride (Millipore, Bedford, MA, USA) membranes under 100V for 2 hrs. After blocked with Tris buffered saline containing 5%(w/v) bovine serum albumin and 0.1% Tween-20, the memberane was incubated with primary antibodies (Table S1) incubated overnight at 4°C followed by Alexa Fluor 488 donkey anti-rabbit or anti-mouse IgG secondary antibody (1:5000, Invitrogen, Eugene, OR, USA) for 1 hr in the dark room. Then membranes were scanned using Typhoon trio (GE Healthcare). The optical densities of all protein bands were analyzed using IMAGEQUANT 5.2 software (GE Healthcare). All samples were run on the same gel. The protein bands were rearranged solely to ease comparison in figures.

Immunofluorescence staining and confocal microscopy

For immunofluorescent staining of brain sections, animals were anesthetized and perfused transcardially with 0.9% saline followed by 4°C PFA in PBS (pH 7.4), then fixed with 4% PFA for 24 hrs. Free floating 30 um sections were cut on a cryostat and stored in antifreeze solution at -20°C. Sections were incubated in blocking solution containing 5% horse serum (Sigma, St. Louis, MO, USA) and 0.3% Triton X-100 in PBS for 2 hrs at RT followed by incubation in primary antibodies (Table S1) at 4°C overnight. The next day, sections were washed with PBS and incubated for 1 hr at RT (light shielded) in secondary antibody (Alexa Fluor 647 donkey anti-rabbit or anti-mouse IgG, 1 : 200, Invitrogen, Eugene, OR, USA) and cover-slipped with 1 drop of Vectashield mounting medium with 4', 6-diamidino-2-phenylindole (DAPI, Vector Laboratories, Burlingame, CA, USA).

For primary neuron cultures, immunofluorescence staining was performed in 24-well plates. Cells were fixed using 4% PFA in PBS for 10 mins at room temperature and washed three times with PBS. Cells were incubated in blocking solution containing 5% horse serum (Sigma) and 0.1% Triton X-100 in PBS for 2 hrs at room temperature followed by incubation with primary antibodies at 41°C overnight. The next day cells were washed with PBS and incubated for 1 hr at room temperature (light shielded) with secondary antibody (Alexa Fluor 647 donkey antirabbit or antimouse IgG, 1:200; Invitrogen, Eugene, OR, USA) and coverslipped with one drop of Vectashield mounting medium with 40, 6-diamidino-2-phenylindole (DAPI; Vector Laboratories, Burlingame, CA, USA).

Immunofluorescent staining was examined using a Zeiss confocal microscope (Zeiss LSM 510, Thornwood, NY, USA).

Transmission electron microscopy (TEM)

Each sample was placed in a droplet of 2.5% glutaraldehyde in PBS buffer and fixed overnight at 4°C. The exosome samples were rinsed 3 times in PBS for 10 mins each and then fixed in 1% osmium tetroxide for 60 mins at room temperature. Then, the samples were embedded in 10% gelatin, fixed in glutaraldehyde at 4°C and cut into small blocks (less than 1 mm^3). The samples were dehydrated in increasing concentrations of alcohol, placed in propylene oxide and infiltrated with increasing concentrations of Quetol-812 epoxy resin mixed with propylene oxide for 3 hrs per step. Finally, the samples were embedded in pure, fresh Quetol-812 epoxy resin, which was allowed to polymerize at 35°C for 12 hrs, 45°C for 12 hrs, and 60°C for 24 hrs. Ultrathin sections were cut using a Leica UC6 ultra-microtome and stained with uranyl acetate for 10 mins and lead citrate for 5 mins at room temperature. The samples were then observed with a transmission electron microscope (Nano Sight Ltd., Amesbury, UK) at a voltage of 110 kV.

Supplementary Tables

Table I. Antibodies, their concentrations and manufacturers in the present studies

**Antibodies**	**Source**	**Dilutions**	**Manufacturer**	**Catalog#**
pmTOR (Ser 2448)	Rabbit	1:1 000	Cell signaling	2971
mTOR	Rabbit	1:1 000	Cell signaling	2983
pS6K p70 (Ser 371)	Rabbit	1:500	Cell signaling	9208
S6K p70	Rabbit	1:500	Cell signaling	9202
pAkt (Ser 473)	Rabbit	1:1 000	Cell signaling	9271
Akt	Rabbit	1:1 000	Cell signaling	9272
pPRAS40 (Thr 246)	Rabbit	1:1 000	Cell signaling	2997
PRAS40	Rabbit	1:1 000	Cell signaling	2691
p53	Rabbit	1:1 000	Cell signaling	2527
LC3 II	Rabbit	1:1 000	Cell signaling	3868
β-actin	Mouse	1:300	Sigma	A-5441
